# Acute and Short-Term Effects of Dry Needling in Patients with Chronic Nonspecific Low Back Pain and Hamstring Tightness: A Pilot Study

**DOI:** 10.1155/2021/7259956

**Published:** 2021-11-20

**Authors:** Mahnaz Bazzaz-Yamchi, Soofia Naghdi, Amin Nakhostin-Ansari, Monavar Hadizadeh, Noureddin Nakhostin Ansari, Ehsan Moghimi, Scott Hasson

**Affiliations:** ^1^Department of Physiotherapy, School of Rehabilitation, Tehran University of Medical Sciences, Tehran, Iran; ^2^Research Center for War-affected People, Tehran University of Medical Sciences, Tehran, Iran; ^3^Sports Medicine Research Center, Neuroscience Institute, Tehran University of Medical Sciences, Tehran, Iran; ^4^Department of Physiotherapy, School of Rehabilitation, Shahid Beheshti University of Medical Sciences, Tehran, Iran; ^5^Department of Physical Therapy, Augusta University, Augusta, Georgia, USA

## Abstract

**Background:**

Chronic nonspecific low back pain (LBP) is one of the common health issues. Hamstring tightness contributes to the development of LBP. This study aimed to investigate the acute and short-term effects of deep dry needling (DN) in patients with chronic nonspecific LBP and hamstring muscle tightness.

**Methods:**

A single-group pretest-posttest clinical study design was followed. The outcome measures were the visual analog scale (VAS), passive knee extension (PKE) test, finger-floor distance (FFD) test, and functional rating index (FRI). Patients underwent one session of deep DN of three points on both hamstring muscles, each point for one minute. Patients were assessed before (T0), immediately after (T1), and one week after DN (T2). The FRI was assessed at T0 and T2.

**Results:**

Ten women with a mean age of 21.1 years (SD = 1.6) participated in the study. Significant large effect sizes in VAS pain reduction (*d* = 1.25) and PKE hamstring tightness were obtained (hamstring: right, *d* *=* 0.82; left, *d* = 0.88) at T2. Medium effect sizes were obtained for FFD (*d* = 0.45) and FRI (*d* = 0.72) at T2.

**Conclusion:**

A single session of deep DN improved pain and function and increased hamstring flexibility. This pilot study supports the use of DN in patients with LBP and hamstring tightness; however, future research with a rigorous study design of randomized controlled trial is required to confirm the findings. This trial is registered with IRCT20180511039612N1.

## 1. Introduction

Low back pain (LBP) is one of the common health conditions, with a prevalence of 1.4–20% in developed countries and a global prevalence of 9.4% [1, 2]. In Iran, the prevalence of LBP is higher than average, and about 27% of Iranian adults suffer from chronic LBP [3]. LBP leads to higher years lived with disability than other conditions globally [2]. In most cases, no underlying pathological condition can be found as the cause of LBP, called nonspecific LBP [4].

Muscle tightness contributes to musculoskeletal conditions. Previous studies have found that hamstring tightness is a contributing factor to LBP [5]. Tightness of hamstring muscles in patients with LBP influences lumbar pelvic rhythm [6, 7] and is associated with severe lumbar pain and changes in the sagittal curvature of spine [8]. Therefore, physiotherapy interventions are often applied to target hamstring muscle tightness in patients with LBP.

Dry needling (DN) is one of the physiotherapy interventions, which has been utilized in patients with LBP [9]. In a clinical trial, 56 male patients with chronic LBP were included and assigned into two groups, DN group (*n* = 29) and control group (*n* = 27); both groups had received 8 weeks standard rehabilitation (physiotherapy, occupational therapy, and exercise therapy) before entering the DN trial. Authors found that the DN group improved significantly compared to the control group after treatment and at follow-up [10]. A systematic review and meta-analysis of 11 randomized controlled trials (RCTs) and 802 patients investigated the DN of myofascial trigger points (MTrPs) in patients with LBP and concluded that the DN of MTrPs is effective in reducing the LBP severity and recommend it for use in LBP especially in combination with other treatments [11]. A systematic review and meta-analysis of 16 RCTs to investigate the effects of DN for LBP concluded that DN is effective in improving LBP pain and disability [12]. Furthermore, a recent scoping review concluded that DN is effective in spine-related conditions including LBP [13]. To date, no study has investigated the effects of DN in patients with LBP and hamstring tightness. Therefore, the aim of the present pilot study was to investigate the acute and short-term effects of DN on pain, hamstring muscle tightness, and function in patients with chronic nonspecific LBP. This pilot study was designed to justify the protocol in patients with chronic LBP and hamstring muscle tightness and to determine the power in order to better develop a larger more robust randomized clinical trial.

## 2. Methods

### 2.1. Design

This pilot study used a single-group pretest-posttest clinical design. The study was conducted in the Physiotherapy Clinic of Tehran University of Medical Sciences (TUMS). The Ethical Committee of TUMS approved the study protocol (Code: IR.TUMS.FNM.REC.1397.056). Study objectives were explained to the patients, and written informed consent was obtained from them.

### 2.2. Participants

Women staff and students of School of Rehabilitation, TUMS, were recruited for the study. Inclusion criteria were as follows: (1) having LBP for at least12 weeks, (2) reduction in the passive knee extension test of ≥25°, (3) severity of the current back pain between 3 and 7 on the VAS, and (4) giving consent to participate in the study. The exclusion criteria were as follows: (1) contraindications for DN, (2) the history of fracture in the lumbar or femoral regions, (3) inability to perform the assessments, and (4) acute recurrence of LBP.

Patients were assessed regarding the eligibility criteria before they entered the study. The same orthopedic physician examined the patients for eligibility and nonspecific LBP diagnosis; however, a physiotherapist also reassessed the patients for eligibility at baseline and before delivering DN. Patients who fulfilled the eligibility criteria underwent one session of DN of the hamstring muscles.

### 2.3. Procedure

The characteristics of age, weight, height, BMI, and LBP duration were recorded. The assessor was an experienced physiotherapist who applied the outcome tests randomly. Another physiotherapist who was experienced using DN and blinded to the results of assessment treated the patients. The assessments were performed before DN (T0), immediately after DN (T1), and one week after the DN (T2). This study was conducted at the Physiotherapy Clinic of TUMS, Tehran, Iran.

### 2.4. Intervention

The physiotherapist delivered the deep DN using disposable sterilized needles (0.25^*∗*^60 mm; DongBang AcuPrime Ltd., Korea). The patients were positioned in prone. DN was performed on three points on the hamstrings for both lower limbs, each point for one minute, using the fast in-fast out cone-shaped technique. As used previously [14, 15], we considered a line between the ischial tuberosity and the fibular head for needling of the long head (60% of the line) and short head (30% of the line) of biceps femoris muscle. The needling of semitendinosus and semimembranosus muscles was performed at a point of 60% of a line from the ischial tuberosity to the medial epicondyle of femur.

### 2.5. Outcomes Measures

The visual analog scale (VAS), passive knee extension (PKE) test, finger-floor distance (FFD) test, and functional rating index (FRI) were the outcome measures to assess the severity of pain, flexibility of hamstring muscle, lumbopelvic mobility, and functional disability.

#### 2.5.1. Visual Analog Scale

We used the VAS to measure the severity of pain at rest. The patients were asked to determine the severity of pain on a 10 cm horizontal line from “0” (no pain) to “10” (most severe pain) [16].

#### 2.5.2. Passive Knee Extension Test

The hamstring tightness for both legs was randomly assessed using the PKE test. Patients were positioned in supine on a firm bed. The contralateral leg was fixed to the table in extension by a strap. The hip and knee of the tested leg was positioned in 90°-90° flexion. Then, the knee was passively extended to the maximal tolerable stretch point reported by the patient; the knee angle was measured at this point using a goniometer [14, 15]. The lower degrees of knee extension were indicative of greater hamstring tightness [17].

#### 2.5.3. Finger-Floor Distance Test

The FFD test was used to assess lumbopelvic mobility. The patients were asked to stand without shoes or socks (bare feet) on the ground while keeping their legs close together. They were asked to bend forward as much as they could and to reach their fingers to the floor without flexing their knees. The distance between the tip of the right middle finger and the floor was measured as the FFD score using a standard tape. Higher distances were indicative of greater hamstring tightness and limited lumbopelvic mobility [18].

#### 2.5.4. Functional Rating Index

We used the Persian version of the self-report FRI to assess disability. This questionnaire consists of 10 items, each item is rated on a Likert scale from “0” (no pain or disability) to “4” (worst possible pain or inability to function). The total score is the sum of all items' scores, ranging between “0” and “40” expressed in percentage disability of “0%” (no disability) and “100%” (severe disability). Persian FRI is a reliable (excellent internal consistency and test-retest reliability) and valid (correlated to the Roland-Morris Disability Questionnaire, Oswestry Disability Questionnaire, Quebec Back Pain Disability Scale, and Numerical Rating Scale) tool for assessing LBP-related disability [19].

### 2.6. Data Analysis

The data analysis was carried out using SPSS software for Windows 22.0 (SPSS Inc., Chicago, IL). We calculated the mean and standard deviation (SD) for continuous variables and number and percentage for categorical variables. The Kolmogorov–Smirnov (KS) test was employed to assess whether the variables were normally distributed. One-way repeated measure of analysis of variance (ANOVA) with time as within-subject factor measured at three time points was used. Bonferroni adjustments were applied for post hoc paired comparisons of testing time points. Mauchley's test was used to analyze the homogeneity of variances. The paired *t*-test was used to analyze the changes of FRI scores. Cohen's d was calculated to determine the magnitude of the DN effect defined as small <0.50, moderate 0.50–0.80, and large ≥0.80 [20]. *P* ≤ 0.05 was interpreted as statistically significant.

Before we began the current pilot study, we conducted a power analysis for the future larger randomized, controlled trial. With ANOVA, repeated measure, between factors as the statistical test, effect size of 0.5, significance level of 0.05, power 0.80, two groups (DN and sham DN), and 3 measurements, a total sample size of 24 patients would provide adequate power to detect meaningful differences between groups.

## 3. Results

Table 1 provides the demographic characteristics of the patients. Ten women with a mean age of 21.1 years (SD = 1.6) participated in the study. Patients had LBP for a mean duration of 3.9 years (SD = 2.25).

### 3.1. Visual Analog Scale

Table 2 presents the outcomes for all tests. Repeated measure ANOVA with sphericity assumption (*p*=0.16) revealed significant decreases in the pain severity after DN (*F*_(2, 18)_ = 22.56, *p* < 0.001). Pairwise comparisons using Bonferroni showed significant reductions in pain severity at T1 (*p*=0.01) and T2 (*p*=0.001). The reduction in pain severity at T2 was significant compared to that at T1 (*p*=0.008).

### 3.2. Passive Knee Extension Test

Repeated measure ANOVA with sphericity assumption (*p* > 0.05) revealed significant increases in both the right (*F*_(2,18)_ = 7.58, *p*=0.004) and left (*F*_(2,18)_ = 5.68, *p*=0.01) PKE after DN at T1 and T2 (Table 2). The increases in PKE of the hamstring muscle of right and left lower extremity remained the same at T2 compared to that at T1 (*p*=1.0).

### 3.3. Finger-Floor Distance Test

Repeated measure ANOVA with sphericity assumption (*p*=0.07) revealed significant improvement in FFD after DN (*F*_(2,18)_ = 9.18, *p*=0.002). Pairwise comparisons using Bonferroni showed significant improvements in FFD at T1 (*p*=0.01) and T2 (*p*=0.04). The improvement in FFD remained the same at T2 compared to that at T1 (*p*=0.94).

### 3.4. Functional Rating Index

The FRI scores significantly improved at T2 one week after DN compared to that at T0 (*t* = 2.91, df = 9, *p*=0.01).

## 4. Discussion

The results of our pilot study showed that a single bout of DN was an effective intervention to decrease pain, improve hamstring flexibility, and improve functional status in patients with chronic nonspecific LBP. Previous studies revealed that DN is effective for pain relief, improving joint range of motion (ROM), muscle strength, and coordination [21, 22]. Most of the studies, however, have focused on the effects of DN on pain, but a limited number of studies have evaluated the effects of DN on flexibility in patients with LBP [23]. In the present study, the effects of DN on pain severity of LBP patients with hamstring tightness have been investigated. Patients with LBP and hamstring tightnesss experienced significant pain reduction with improvement in hamstring flexibility and function. As far as we know, this is the first study that investigated the effects of DN on hamstring tightness in a patient group with chronic LBP.

The effect size of DN in pain reduction immediately after treatment was moderate and large at seven days follow-up. In order to be clinically significant, a 20% change in VAS should be reported for individuals with chronic LBP [24, 25]. In this study, the changes in pain severity were 18% and 48% immediately after DN and at one-week follow-up, respectively. This indicates that the reduction of pain after treatment with DN was clinically relevant in this small sample of patients with chronic nonspecific LBP. A Cochrane systematic review of 35 randomized controlled trials with 2861 patients concluded that DN is a useful modality for chronic LBP [9]. Another systematic review and meta-analysis of 16 randomized controlled trials (RCTs) to investigate the safety and effectiveness of DN for the management of LBP found that DN was an effective intervention for LBP in reducing pain and disability compared to the acupuncture and sham needling, and its effectiveness was the same as the acupuncture at follow-up [12]. Large effect size in pain reduction one week after DN that was much greater than the minimally clinically important change (MCIC) indicates that with passage of time, pain reduction continues. It follows that physiotherapists should expect significantly greater reduction in pain severity beyond the immediate effects of DN at least one week later in patients with chronic LBP.

Hamstring muscle tightness has been demonstrated to be higher in patients with LBP than healthy adults [26, 27]. Hamstring muscle tightness can restrict the lumbopelvic ROM which in turn results in posterior tilt and decrease in the lumbar curve [27–29], contributing to development of LBP [30]. Flexibility of the hamstring muscles are thus important to reduce the load on the lumbar area during daily activities [30]. In the current study, the hamstring flexibility improved with effect sizes ranging from moderate immediately after DN to large at one-week follow-up in terms of PKE. Improvements in PKE indicate increases in hamstring extensibility and knee ROM. Improvements in hamstring flexibility is in line with the recent findings on the effects of DN on hamstring flexibility in healthy subjects [14, 15]. In a single-blinded pilot study, significant large effects sizes were revealed for hamstring muscle compliance and flexibility as well as stretch tolerance [15]. A further single-blinded RCT involving two groups receiving DN or static stretch in healthy subjects with hamstring tightness found improvements in the active knee extension test, hamstring muscle compliance, passive peak torque, and stretch tolerance which were better for the DN group compared to the static stretch group [14]. The current study demonstrated greatest gains in PKE ROM at one-week follow-up.

Flexibility of the trunk is usually the goal of physiotherapy programs in patients with LBP. We used the FFD as a relevant outcome measure to assess total mobility of the spine and the lumbopelvic rhythm in our patients with chronic LBP. In the current study, small effect sizes were obtained for the FFD despite clinically relevant improvements of 29% immediately after DN and 48% at one-week follow-up. Again, greater improvement occurred at one-week follow-up confirming the time required for DN effects to be optimized [15]. The possible reasons for the small effect sizes observed for the FFT might be that the sample size was small, and the patients included in this study were active young women, so forward bending flexibility was not considerably restricted; albeit, we expected more restriction in FFD scores as patients with chronic LBP often present with lower scores at baseline visit [31, 32]. Nevertheless, improvements of ≥30% occurring after DN are clinically relevant. Further study with a larger sample of patients with LBP and both gender of various ages and activity level is required to verify the findings.

The FRI as a measure of function was improved 30% in this sample with chronic nonspecific LBP. Although a moderate effect size (*d* = 0.72) was obtained, improvements were greater than MCIC of 10.63% reported for the Persian FRI in patients with chronic LBP [33]. This finding suggests that the patients perceived the improvements in function as important and worthwhile. Improvements in function could be due to the large analgesic effects of DN and increases in hamstring flexibility and trunk mobility.

The current pilot study was primarily conducted to provide information on the feasibility, treatment effect sizes on outcomes, and data for power determination to design the future randomized trial. The present pilot study provides preliminary evidence on efficacy on the pain, hamstring flexibility, and disability in patients with chronic LBP with hamstring tightness. However, our sample size estimation for two groups with sham DN as a control group shows a total sample size of 24 patients to be assigned equally for two groups. Hence, the sample size for this pilot study was small to draw definite conclusion on the efficacy of DN in chronic LBP patients with hamstring tightness. So, the future main rigorous study should be undertaken to eliminate the potential biases and to answer the research question justified by the positive outcomes found in this pilot study. Nevertheless, despite the small sample size of the present pilot study and the lack of a control group, its results support the DN intervention for alleviating pain and disability in patients with chronic LBP and hamstring tightness.

The current study has limitations. First the sample size was small. Second, only women were included. A convenient group of students and office workers was included. Chronic low back pain is common even in young women individuals [34], especially those students and office workers that sit for a long time [35]. Future studies need to include chronic LBP patients of both genders with a wide range of ages. Third, this was a pilot single-group study without a comparative group. Fourth, the short-term effects of DN were evaluated. Fifth, DN was applied as a single therapy; we aimed to evaluate the role of only DN in patients with LBP. Dry needling applied as an element of a multimodal physiotherapy program may enhance the improvements especially in combination with exercise therapy. Sixth, we used DN for just one treatment session. The effectiveness of DN may vary with increases in the number of treatment sessions. Future studies should compare the various treatment frequencies in patients with chronic LBP. Nevertheless, considering the time and costs associated with common multiple sessions of treatment in clinical practice, a single session of DN for a total 3 minutes of each hamstring muscle with large effect sizes on outcomes is worthwhile.

## 5. Conclusion

A single session of dry needling used in this study of patients with chronic LBP and hamstring tightness improved pain and function and increased hamstring flexibility. The usefulness of DN applied as a stand-alone therapy suggests a promising intervention in clinical practice for patients with chronic nonspecific LBP and hamstring tightness. The current study was a pilot single-group clinical trial without the sham control group that may make the dry needling appear more effective intervention. Further rigourous double-blinded randomized clinical investigations with long-term follow-up are warranted to confirm the findings.

## Figures and Tables

**Table 1:** Demographic characteristics of the participants (*n* = 10).

	Mean ± SD^‡^	Minimum	Maximum
Age (year)	21.1 ± 1.6	19	24
BMI^*∗*^ (kg/m^2^)	24.31 ± 3.54	20.30	32.30
Duration of LBP⁑ (year)	3.9 ± 2.25	1.5	9.0

^*∗*^BMI, body mass index; ⁑LBP, low back pain; ^‡^SD, standard deviation.

**Table 2:** Results of outcomes before, after, and one week after dry needling (*n* = 10).

			Mean ± standard deviation
Variable	Before	Immediately after	One week after
**VAS** ^*∗*^	4.76 ± 1.38	3.89 ± 1.83, *d*^*⁂*^ = 0.54	2.49 ± 2.16, *d* = 1.25
PKE^†^ right limb (degree)	43.60 ± 8.64	50.16 ± 9.76, *d* = 0.71	50.73 ± 8.82, *d* = 0.82
PKE^†^left limb (degree)	41.87 ± 9.83	48.5 ± 12.58, *d* = 0.59	50.0 ± 8.65, *d* = 0.88
FFD^⁑^ (cm)	8.25 ± 5.22	5.86 ± 6.57, *d* = 0.40	5.24 ± 8.0, *d* = 0.45
FRI^‡^	29.75 ± 11.87	—	21.25 ± 11.62, *d* = 0.72

^*∗*^VAS, visual analog scale; ⁑FFD, finger-floor distance; ‡PKE, passive knee extension; ‡FRI, functional rating index; ⁂Cohen's d.

## Data Availability

The datasets used and/or analyzed in this study are available from the corresponding author upon request.
